# The Prevalence of and Factors Associated with Prediabetes Among Adolescents in Central Sudan: A Community-Based Cross-Sectional Study

**DOI:** 10.3390/children12111447

**Published:** 2025-10-24

**Authors:** Walaa M. Alsafi, Abdullah Al-Nafeesah, Ashwaq AlEed, Ishag Adam

**Affiliations:** 1 Faculty of Medicine, Gadarif University, Gadarif 32211, Sudan; 2 Department of Pediatrics, College of Medicine, Qassim University, Buraydah 52571, Saudi Arabia; 3 Department of Obstetrics and Gynecology, College of Medicine, Qassim University, Buraydah 52571, Saudi Arabia

**Keywords:** prediabetes, adolescent, prevalence, female, HbA1c, age

## Abstract

**Background:** Prediabetes is a significant precursor to type 2 diabetes mellitus (T2DM) and its well-known complications. In Sudan, data on the epidemiology of prediabetes among adolescents are scarce, especially in the central region. Thus, this study aimed to determine the prevalence of and factors associated with prediabetes among adolescents in central Sudan. **Methods:** This community-based cross-sectional study was carried out in East Gezira, central Sudan, from April to June 2025 and included 379 adolescents. Sociodemographic characteristics, anthropometric data (body mass index and BMI-z-score), and clinical information were gathered. Prediabetes was defined as a glycated hemoglobin (HbA1c) level in the range of 5.7% to 6.4%. Multivariate binary analysis was applied to identify the factors associated with prediabetes. **Results:** The median (interquartile range, IQR) age and BMI-z-score of the 379 enrolled adolescents were 14 (12–16) years and −1.4 (−2.1–−0.39), respectively. Sixty-four (17.0%) adolescents had prediabetes. Univariate analysis did not reveal any significant associations between the investigated factors and prediabetes. However, multivariate binary analysis showed that being female was independently associated with prediabetes among adolescents (adjusted odds ratio, AOR = 1.80, 95% confidence interval, CI 1.01–3.18). Age, BMI z-score, parent education, and occupation were not associated with prediabetes. **Conclusions:** The prevalence of prediabetes among adolescents in central Sudan is substantial, highlighting a potential future surge in T2DM. The finding that female adolescents are at a higher risk underscores the need for targeted, gender-sensitive screening and preventive health programs to mitigate the progression from prediabetes to T2DM.

## 1. Introduction

There is an increasing global trend in both prediabetes and diabetes mellitus (DM) in adolescents, including those in sub-Saharan Africa (SSA) [1,2,3,4]. The American Diabetes Association defines prediabetes as glycated hemoglobin (HbA1c) levels ranging from 5.7% to 6.4% [5]. Prediabetes, both in the general population and specifically in adolescents, has recently received increased attention for several reasons, including the global increase in type 2 diabetes mellitus (T2DM) in adolescents, with SSA being no exception [2,3,4]. Ng and Chan reported that youth-onset DM is particularly concerning due to its more aggressive progression than adult-onset diabetes, resulting in a faster decline in the body’s ability to produce insulin and causing accelerated end-organ damage [6]. Unless appropriate preventative measures are taken, this global increase in prediabetes in adolescents will lead to a rise in the incidence and prevalence of T2DM onset in adulthood [6,7,8]. In addition, a key problem of prediabetes arises from its progression to T2DM [9,10] and its well-known complications, including diabetic nephropathy, cardiovascular diseases, microalbuminuria, and diabetic retinopathy [6,11,12,13,14,15]. Unfortunately, such complications, including cardiovascular diseases, may place a significant burden on already fragile health systems, such as those in SSA, including Sudan [3,8]. However, it is possible to slow or halt the progression from prediabetes to frank DM [9,10] or even reverse prediabetes [3,16].

According to the International Diabetes Federation (IDF), approximately 90% of people with undiagnosed DM live in low- and middle-income countries [15]. Therefore, any approach to preventing DM and its associated complications is recommended in these limited-resource settings [3,6]. Different prevalence rates have been reported regarding prediabetes among adolescents in populations such as SSA [1,17], including Sudan [18]. High prevalence rates of prediabetes in adolescents have been reported in India (16.2%) [19] and the United States (18%) [8]. In contrast, Nigeria has a low prevalence rate of prediabetes in adolescents, ranging from 4% to 9.4% [4,20].

The literature reveals several factors associated with prediabetes in adolescents, including age [4,8], sex [8,21], low parental education level [22], parental employment [4,18,23], tobacco use [24], a family history of DM [4,21], and a high body mass index (BMI) [8,20,25].

Although existing research has addressed prediabetes among adults in Sudan [26,27], little is known about the epidemiology of prediabetes in Sudanese adolescents [18]. According to the World Health Organization (WHO), more than 20% of Sudan’s population is composed of adolescents [28]. The ongoing war in Sudan is negatively impacting the health of children and adolescents, especially those with chronic diseases such as DM who need adequate storage conditions for their medications [29,30,31]. In addition, there is low awareness of prediabetes and DM among the Sudanese population, even among healthcare professionals [32,33]. The authors’ prior studies in eastern Sudan reported high prevalence rates of both prediabetes among adolescents and DM and its complications among adults [18,34,35]. To increase proactivity, a clear map of prediabetes prevalence and associated factors is needed across different regions of Sudan, particularly at the community level. Thus, this study aims to investigate the prevalence of and factors associated with prediabetes among adolescents in East Gezira, central Sudan.

## 2. Materials and Methods

### 2.1. Study Design and Setting

This community-based cross-sectional study was conducted from April to June 2025 among adolescents in Elrikieb, East Gezira, central Sudan. Gezira State is located in east-central Sudan, lying between the Blue and White Nile Rivers. Elrikieb was chosen because it is composed of four sub-villages whose inhabitants have similarities with those of the overall Gezira State. The authors strictly adhered to the Strengthening the Reporting of Observational Studies in Epidemiology (STROBE) guidelines [36].

### 2.2. Study Population and Sampling

A total of 379 adolescents were recruited from the community using a systematic approach. A multistage stratified random sampling approach was employed to select potential participants. Within each selected sub-village, households were randomly assigned (lottery method) in proportion to the sub-village’s population and the desired sample size; one adolescent from each household was invited (lottery method) to participate. If there were no adolescents in the selected household or if they refused to participate, the next household was chosen.

### 2.3. Inclusion and Exclusion Criteria

Before data collection, informed consent was obtained from the parents or guardians. Based on the WHO definition of adolescents [28], all individuals aged 10–19 years were included in the present study, with those younger than 10 or older than 19 years being excluded, along with those who did not consent to participate in the study. All recruited participants were apparently healthy (based on self- and parental report), and adolescents who were sick, were known diabetics, or were pregnant as well as nursing adolescent girls were excluded from the study.

### 2.4. Sample Size Calculation

The desired sample size was computed using OpenEpi Menu software Version 3.01 [37]. The authors assumed that 35% of adolescents would have prediabetes. This assumption was based on the authors’ prior study in Eastern Sudan, which revealed that 32.6% of adolescents had prediabetes or DM [20]. Thus, a sample size of 379 adolescents was computed for this study to detect a 5% difference at α = 0.05 with 80% power. Moreover, the authors assumed that 10% of adolescents would not respond or would have incomplete data.

### 2.5. Study Variables and Measures

Following a thorough review of the literature, including research in Sudan [4,8,18,20,21,25], we developed a questionnaire designed to collect sociodemographic data, including age in years, sex (male or female), parental educational levels (<secondary or ≥secondary), mother’s occupational status (housewife, employed, or none), father’s occupational status (working or none), tobacco use (yes or no), family history of DM (yes or no), and presence of a family member who smokes (yes or no). Anthropometric measurements such as weight and height were collected. The WHO reference was used to compute the body mass index (BMI) z-score [38].

After the participants and their guardians agreed to participate and signed an informed consent form, the selected adolescents were approached by trained research assistants. The adolescents were informed about the study’s objectives and all necessary information, including the voluntary nature of their participation, their right to withdraw at any time without having to provide a reason, and the measures taken to ensure their privacy, confidentiality, and safety, such as excluding personal identifiers during data collection. In this study, the primary outcome was prediabetes, and the independent variables were sociodemographic data and BMI z-score. To achieve the study’s objective, a few newly diagnosed cases of T2DM (*n* = 12) were excluded from the model.

### 2.6. Blood Sample Processing

Under septic conditions, 3 mL of blood was taken in an ethylenediaminetetraacetic acid tube for HbA1c analysis. An Ichroma machine was used to measure HbA1c according to the manufacturer’s instructions (Republic of Korea), as detailed in the authors’ prior work [34]. Prediabetes can be defined as an HbA1c level in the range of 5.7–6.4%, and T2DM can be defined as an HbA1c level of 6.5% or greater [5]. HbA1c has been used as a diagnostic tool for prediabetes/DM among children and adolescents in different contexts, including Sudan [18,22,39]. In this study, prediabetes and DM were diagnosed using HbA1c as it is not influenced by the last meal, which makes it more suitable in limited-resource settings. Furthermore, Ghaddar et al. reported that analyses including fasting plasma glucose and HbA1c yielded similar findings in children and adolescents [22]. It is worth mentioning that other studies have used the WHO’s definition for prediabetes, namely, impaired fasting blood glucose (110–125 mg/dL) and impaired glucose tolerance (2 h glucose 140–199 mg/dL) [2,21].

### 2.7. Statistical Analysis

The data were entered into SPSS statistical software for Windows (version 22.0; SPSS Inc., New York, NY, USA) for analysis. Continuous data, such as age and BMI z-score, were tested for normality using the Kolmogorov–Smirnov test and were found to be non-normally distributed. Consequently, they are expressed as medians (interquartile ranges, IQRs). Initially, univariate binary analysis was performed with HbA1c as the categorical dependent variable (prediabetes vs. nondiabetes) and sociodemographic data—such as age, sex, parents’ education, parents’ occupation, tobacco use, and family history of DM—as the independent variables. All variables from the univariate binary analysis were included in a multivariate analysis to adjust for covariates (backward stepwise selection using likelihood ratio). Multicollinearity was checked using the Variance Inflation Factor (VIF, more than 5) and was not detected. Adjusted odds ratios (AORs) and 95% CIs were computed as they were applied. A two-sided *p*-value less than 0.05 was considered statistically significant.

## 3. Results

A total of 379 adolescents were enrolled in this study, including 194 females (51.2%) and 185 males (48.8%). The median (IQR) age and BMI z-score were 14.0 (12.0–16.0) years and −1.4 (−2.1–−0.39), respectively. In total, 58 mothers (15.3%) and 103 fathers (27.2%) had secondary education or above. Most of the mothers were housewives (*n* = 340, 89.7%), and 333 (87.9%) of the fathers were employed. Nearly one-fifth (17.9%) of the adolescents had a family history of DM in a first-degree relative. The median (IQR) HbA1c was 5.4% (5.1–5.5%). One-fifth (*n* = 76, 20%) of these adolescents had prediabetes or T2DM. Among the 379 participants, 303 (80.0%) had no DM, 64 (17.0%) had prediabetes, and 12 (3.0%) had DM. The prevalence of prediabetes was higher in female adolescents than in male adolescents (*n* = 37, 57.8% vs. *n* = 27, 42.2%); however, this difference was not significant (Table 1). Females had a higher median BMI z-score (IQR) of −0.8 (−1.8–0.3) compared to their male counterparts at −1.6 (−2.4–−1.0), *p* < 0.001.

In the univariate binary analysis, none of the investigated factors (age, gender, BMI z-score, tobacco use, parent education and occupation, and family history of DM), were found to be associated with prediabetes. However, in the multivariate binary analysis, being female was found to be a risk factor for prediabetes (AOR = 1.80, 95% CI 1.01–3.18) (Table 2).

## 4. Discussion

This study is the first to provide community-based data on the prevalence of prediabetes and its associated factors in adolescents in central Sudan. This prevalence (17.0%) represents a significant public health concern, highlighting the substantial burden of this precursor condition among adolescents in central Sudan, and it is comparable to the high rates observed in eastern Sudan (30.0%) [18] and other countries such as India (16.2%) [19] and the United States (18.0%) [8]. This high prevalence of prediabetes and DM supports the increasing global trend of prediabetes and DM among adolescents, including in SSA [2,3,4]. This suggests that a considerable number of adolescents in central Sudan are at risk of developing T2DM and its related complications, underscoring the urgent need for targeted preventive interventions. In addition, the high prevalence of prediabetes (17.0%) observed in this study may explain the elevated prevalence of T2DM among adolescents and adults in different regions [18,26,34,35]. The observed prevalence of T2DM among adolescents in central Sudan (3.0%) was higher than that observed in the United Arab Emirates (0.9%) [23], Cote d’Ivoire (0.4%) [2], and the United States (1.1%) [40].

The most compelling finding of this study is the independent association between the female sex and the approximately twofold higher risk of prediabetes. However, the confidence interval is wide, and the *p*-value is borderline (AOR = 1.80, 95% CI = 1.01–3.18, *p* = 0.045). This finding is noteworthy as previous studies have either found no sex-based difference or have reported higher rates among males [8,18,21]. However, this finding aligns with other studies reporting a higher prevalence of prediabetes in female children and adolescents in specific populations [41,42,43]. The reasons for this gender disparity among adolescents are likely complex and may be rooted in a combination of biological, social, and cultural factors. For instance, specific hormonal changes during puberty, unique dietary practices, physical inactivity, and psychosocial stressors related to gender roles in this cultural context could contribute to increased risk. This finding highlights the critical need for gender-sensitive public health programs that specifically target female adolescents through screening programs and educational initiatives.

Interestingly, this study did not find any significant association between prediabetes and several other factors commonly implicated in the literature, such as age [4,8], low parental education level [22], parental employment [4,18,23], tobacco use [24], family history of DM [4,21], or high BMI [8,20,25]. The findings regarding BMI, which is widely considered a key risk factor for prediabetes globally [8,20,25], are particularly striking. The median BMI z-score in this study population was low (−1.4), which may indicate that obesity is not the primary driver of prediabetes in this specific community. However, in this study, females had a higher median (IQR) BMI z-score than their male counterparts. Such differences in BMI z-score between sexes and the influence of gender on prediabetes require further exploration. In addition, the ongoing war in Sudan and the associated socioeconomic instability may have altered the etiological landscape, with other factors—such as psychosocial stress, dietary shifts toward more processed foods, and disruptions to regular metabolic function—playing a leading role. These factors, which were not comprehensively captured in this study, represent a crucial area for future research. The lack of association with DM family history—a well-established genetic risk factor—was unexpected and may have been due to the underreporting of this condition in a population with low health literacy and in a fragmented healthcare system. Moreover, the absence of an association between these factors and prediabetes may be due to a genuinely low influence in this context, or it may reflect limitations in measurement (e.g., self-reported family history and relatively low BMI z-score overall).

### 4.1. Study Implications

The findings of this study address the urgent need for community-based interventions in central Sudan. With 17% of adolescents having prediabetes in the region, health initiatives should prioritize early screening programs, particularly for females, who were identified as a high-risk group. The findings underscore the importance of promoting healthy lifestyles, including physical activity and balanced nutrition, to prevent the progression from prediabetes to T2DM in this vulnerable population. One practical implication of this study was that the 12 adolescents identified with frank T2DM were advised to seek medical care at the nearest healthcare facility for further evaluation and management.

### 4.2. Strengths and Limitations

This study has several strengths, including its community-based design, which provides a more representative picture of the population than hospital-based studies. This research can add value to the limited number of studies on prediabetes, especially those of adolescents in Sudan [26,27]. Regarding central Sudan, this study provides a vital baseline in a region with no prior data on this topic, enabling future trend analysis and the evaluation of intervention effectiveness. However, this study has some limitations that should be noted to inform future research. The cross-sectional design precludes any inference of causality, and the study was conducted over a short period, meaning it may not capture seasonal variations in glucose regulation. Furthermore, while the authors collected data on several key risk factors, other important variables—such as dietary habits [4], physical activity levels [4,25], and psychological stress—were not assessed in detail. The reliance on self-reported data for some variables, such as family history of DM, is a potential source of bias.

Fasting plasma glucose or oral glucose tolerance test (OGTT) was not assessed. Using HbA1c as the sole diagnostic tool is practical in low-resource settings, but it has its limitations. Anemia, hemoglobinopathies, or nutritional deficiencies may influence HbA1c, which could lead to over- or under-estimation of prevalence.

The calculation assumes a prevalence of 35% based on a previous study in eastern Sudan, but the observed prevalence was much lower (17%). This discrepancy may affect the statistical power of this study.

## 5. Conclusions

This study highlights the high prevalence of prediabetes among adolescents in central Sudan, with the female sex being identified as a significant independent risk factor. These findings are crucial for informing targeted public health interventions aimed at this vulnerable group. Future research is warranted, including longitudinal studies that explore the complex interplay of environmental, social, and biological factors in this unique setting, to better understand the drivers of prediabetes and to develop effective strategies to prevent its progression to type 2 diabetes mellitus (T2DM).

## Figures and Tables

**Table 1:** Sociodemographic and clinical characteristics of the studied adolescents in central Sudan (*n* = 379), 2025.

Variable		Total (*n* = 379)	
		Median	Interquartile range
Age (years)		14.0	12.0–16.0
Body mass index z-score		−1.4	−2.1–−0.39
Glycated hemoglobin %		5.4	5.1–5.5
		Frequency	Percentage
Sex	Male	194	51.2
	Female	185	48.8
Mother’s education	≥secondary	58	15.3
	<secondary	321	84.7
Father’s education	≥secondary	103	27.2
	<secondary	276	72.8
Mother’s occupation	Housewife	340	89.7
	Employed	25	6.6
	None	14	3.7
Father’s occupation	Working	333	87.9
	None	46	12.1
Tobacco use	Yes	14	3.7
	No	365	96.3
Family history of diabetes	No	311	82.1
	Yes	68	17.9
Presence of a smoker in the family	No	263	69.4
	Yes	116	30.6
Diabetes status	None	303	80.0
	Prediabetes	64	17.0
	Diabetic	12	3.0

**Table 2:** Univariate and multivariate binary analyses of the factors associated with prediabetes among adolescents in central Sudan (*n* = 367), 2025.

Variable		Adolescents with Prediabetes (*n* = 64)	Adolescents Without Prediabetes (*n* = 303)	Univariate Analysis		Multivariate Analysis	
		Median (Interquartile Range)		Non–Adjusted Odds Ratio (95% Confidence Interval, CI)	*p*	Adjusted Odds Ratio (95% CI)	*p*
Age, years		14.0 (11.2–16.0)	14.0 (12.0–16.0)	1.01 (0.97–1.05)	0.559	0.93 (0.83–1.05)	0.296
Body mass index z-score		−1.14 (−1.9–−0.3)	−1.45 (−2.1–−0.4)	1.09 (0.96–1.25)	0.172	1.11 (0.96–1.18)	0.155
	Frequency(proportion)						
Sex	Male	27 (42.2)	163 (53.8)	Reference			
	Female	37 (57.8)	140 (46.2)	1.60 (0.93–2.75)	0.093	1.80 (1.01–3.18)	0.045
Mother’s education level	≥Secondary level	10 (15.6)	48 (15.8)	Reference			
	˂Secondary level	54 (84.4)	255 (84.2)	1.01 (0.48–2.13)	0.966	1.90 (0.75–4.82)	0.174
Mother’s occupation status	Housewife	55 (85.9)	273 (90.1)	Reference			
	Employed	7 (10.9)	18 (5.9)	1.93 (0.76–4.84)	0.161	2.52 (0.82–7.71)	0.105
	None	2 (3.1)	12 (4.0)	0.82 (0.18–3.80)	0.807	0.77 (0.16–3.69)	0.747
Father’s education level	≥Secondary level	23 (35.9)	77 (25.4)	Reference			
	˂Secondary level	41 (64.1)	226 (74.6)	0.60 (0.34–1.07)	0.088	0.58 (0.31–1.05)	0.075
Father’s occupation status	Employed	59 (92.2)	264 (87.1)	Reference			
	None	5 (7.8)	39 (12.9)	0.57 (0.21–1.51)	0.263	0.56 (0.21–1.55)	0.270
Family history of diabetes mellitus	No	53 (82.8)	249 (82.20	Reference			
	Yes	11 (17.2)	54 (17.8)	0.95 (0.46–1.95)	0.904	0.85 (0.40–1.83)	0.688
Presence of a smoker in the family	No	46 (71.9)	207 (68.3)	Reference			
	Yes	18 (28.1)	96 (31.7)	0.84 (0.46–1.53)	0.577	0.73 (0.38–1.37)	0.331
Tobacco use	No	61 (95.3)	292 (96.4)	Reference			
	Yes	3 (4.7)	11 (3.6)	1.30 (0.35–4.82)	0.689	1.89 (0.45–7.79)	0.378

## Data Availability

The data of this study will be available from the corresponding author upon reasonable request.

## Abbreviations

The following abbreviations are used in this manuscript:

AOR	adjusted odds ratio
CI	confidence interval
DM	diabetes mellitus
IQR	interquartile range
HbA1c	glycated hemoglobin
US	United States
WHO	World Health Organization
